# Effects of Dance-Based Aerobic Training on Frailty and Cognitive Function in Older Adults with Mild Cognitive Impairment: A Randomized Controlled Trial

**DOI:** 10.3390/diagnostics15030351

**Published:** 2025-02-03

**Authors:** Marcelina Sánchez-Alcalá, Agustín Aibar-Almazán, María del Carmen Carcelén-Fraile, Yolanda Castellote-Caballero, Javier Cano-Sánchez, Alexander Achalandabaso-Ochoa, Juan Miguel Muñoz-Perete, Fidel Hita-Contreras

**Affiliations:** 1 Department of Health Sciences, Faculty of Health Sciences, University of Jaen, 23071 Jaen, Spain; 2 Department of Educational Sciences, Faculty of Social Sciences, University of Atlántico Medio, 35017 Las Palmas de Gran Canaria, Spain; 3 Department of Health Sciences, Faculty of Health Sciences, University of Atlántico Medio, 35017 Las Palmas de Gran Canaria, Spain

**Keywords:** dance-based aerobic training program, frailty, cognitive function, mild cognitive impairment, older adults

## Abstract

**Background/Objectives:** The purpose of this study has been to evaluate the effects of a dance-based aerobic training program on frailty, cognitive impairment, executive functions, and verbal fluency in older adults with mild cognitive impairment. **Methods:** Randomized clinical trial, whose sample was made up of 92 older adults, of which 47 performed rhythmic physical activity for 12 weeks. Data on frailty were collected through FRAIL, cognitive function through the Mini-Mental State Examination (MMSE), cognitive impairment through The Montreal Cognitive Assessment (MoCA), verbal fluency using the Isaac test, and executive functions with the Trail Making Test (TMT). All variables were measured before and after the intervention by an independent researcher blinded to the treatment. **Results:** Participants in the experimental group showed statistically significant improvements in frailty (Cohen’s d = 0.60), cognitive function (Cohen’s d = 0.98), cognitive impairment (Cohen’s d = 1.22), verbal fluency (Cohen’s d = 0.61) and executive functions (Cohen’s d = 0.64). **Conclusions:** This study demonstrated that a 12-week dance-based aerobic training program can significantly reduce frailty and improve cognitive abilities in older adults with mild cognitive impairment. These improvements suggest that the intervention is not only effective in terms of physical health, but also in promoting mental health and general well-being.

## 1. Introduction

Population aging is a global phenomenon that implies important challenges for public health systems [1]. As life expectancy increases, so does the proportion of older adults in the population [2]. This demographic change highlights the need to promote healthy aging and improve the quality of life in this age group, particularly in the face of conditions such as mild cognitive impairment (MCI) [3]. MCI represents an intermediate phase between normal cognitive aging and dementia, characterized by a reduction in memory and other cognitive abilities that do not yet significantly interfere with daily activities [4]. Recent research suggests that the prevalence of MCI is increasing, in part due to increased life expectancy and an aging global population [5,6], and may lead to physical frailty [7], decreased executive functions [8], and impairment of verbal fluency [9], which can significantly reduce the independence and quality of life of the elderly [10].

Frailty is a clinical syndrome characterized by decreased resistance and physiological reserve against stressors, increasing the risk of various adverse events such as falls and hospitalizations [11]. This syndrome is prevalent in the geriatric population and is associated with greater vulnerability to infections, postoperative complications, and mortality [12]. According to Fried et al. [13], the most common criteria for diagnosing frailty include involuntary weight loss, muscle weakness, decreased walking speed, exhaustion, and low physical activity. In addition to these criteria, recent studies have identified other risk factors for frailty, such as chronic inflammation, sarcopenia (loss of muscle mass), and oxidative stress [14]. The assessment and management of frailty have become a priority in geriatric care, given its significant impact on the quality of life and survival of older patients [15].

Executive functions, including planning, problem-solving, and impulse control, are essential for the independent management of daily life and also tend to decline with age [16]. These functions are critical for carrying out complex tasks and for making decisions in everyday situations [17]. Recent studies have shown that aging significantly affects cognitive flexibility, working memory, and inhibition, which are key components of executive functions [18]. The decrease in these capacities can lead to difficulties in organizing activities, time management, and adaptation to unexpected changes, which negatively impact the quality of life and autonomy of older people [19]. Likewise, verbal fluency, which implies the ability to generate words and form fluent sentences, is crucial for effective communication and is another cognitive aspect susceptible to the impact of aging, decreasing with age [20]. This reduction in verbal fluency can make communication difficult, leading to frustration and social isolation in older people. Factors such as reduced processing speed and decreased semantic memory contribute to these difficulties [21].

Physical activity plays a key role in improving both cognitive and physical resilience, being a crucial factor in the maintenance and improvement of cognitive reserve in older adults [22]. Several studies such as that of Moret et al. [23] have shown that regular exercise not only prevents chronic diseases, but that it also powers the brain’s ability to adapt to aging-related challenges, improving areas such as memory, executive functions, and processing speed. In addition, exercise contributes to increased strength and mobility, which translates into a better quality of life and greater independence [24]. In this context, physical activity is set up as a fundamental tool not only for the prevention, but also for the improvement of resilience against cognitive impairment, enabling older people to reduce the risk of cognitive impairment and promote healthy brain aging [25]. Within the spectrum of physical activities, aerobic exercise emerges as particularly beneficial due to its ability to improve both cardiovascular and respiratory health [26]; dance, as a form of aerobic exercise, not only encourages physical activity but also incorporates cognitive and social aspects by requiring memorization of steps, coordination, and rhythm, providing stimuli for the mind and body, promoting improvements in verbal fluency and executive functions, in addition to counteracting cognitive decline and frailty [27].

Therefore, the objective of this study is to evaluate the effects of a dance-based aerobic training program on frailty, cognitive impairment, executive functions, and verbal fluency in older adults with mild cognitive impairment. Based on previous evidence, we hypothesize that aerobic training through dance will not only reduce frailty and cognitive decline, but will also improve executive functions and verbal fluency in participants. This study is expected to contribute to the existing body of knowledge on effective interventions to improve the health and quality of life of older adults.

## 2. Materials and Methods

### 2.1. Research Design and Participants

This study was conducted as a randomized controlled clinical trial between January and April 2024. Prior to any intervention, all participants received comprehensive information about the project and provided written consent to participate. The research was approved by the Ethics Committee of the University of Atlántico Medio (CEI/05-007), adhering to the ethical principles outlined in the Declaration of Helsinki, and was registered under the number NCT06130878.

**Figure 1:**
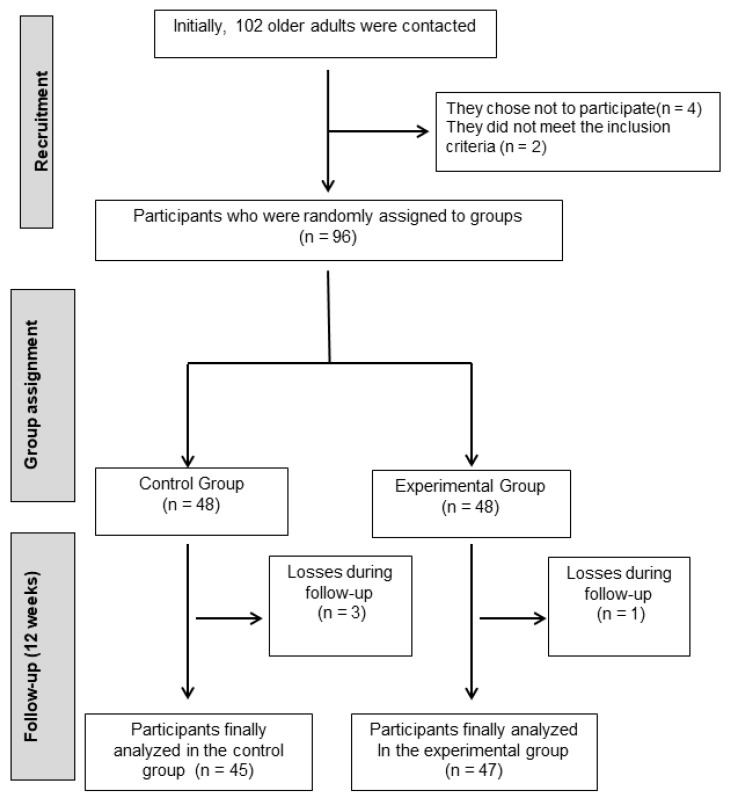
Flowchart of participants in this process.

A total of 102 older adults with mild cognitive impairment were initially approached. Following the selection process, 4 individuals declined to participate, and 2 did not satisfy the inclusion criteria. As a result, 96 participants were successfully enrolled and randomly allocated into two study groups (Figure 1).

Older adults were included in the study if they: (i) were aged 65 years or older; (ii) were not participating in any physical exercise program; (iii) scored below 24 on the MMSE; (iv) demonstrated physical autonomy to engage in the required physical activities; (v) had the ability to understand the study’s instructions, programs, and protocols; and (vi) provided signed informed consent. Conversely, older adults were excluded if they: (i) had any systemic condition (e.g., neurodegenerative, musculoskeletal, or vision impairments) that prevented them from performing the postural balance test or exercise activities; (ii) presented with vestibular disorders or diseases; or (iii) were taking medications that impact the central nervous system, balance, or coordination (e.g., antidepressants, anxiolytics, or vestibular sedatives), including those that enhance cognition or vestibular function.

### 2.2. Randomization

The older adults included in this study were randomly allocated into two groups, an experimental group, and a control group, in a 1:1 ratio. The randomization process was conducted using a computer-generated random number table. Group allocation was managed using sealed opaque envelopes, and this task was handled by an independent entity that was not involved in participant selection, intervention implementation, or data analysis. A total of 48 participants were assigned to the Experimental Group (EG), which participated in a dance-based aerobic training program, while 48 participants were allocated to the Control Group (CG). Participants in the control group were instructed to maintain their usual daily routines and to avoid any formal training programs. Additionally, they received guidance to encourage physical activity.

### 2.3. Intervention

The dance-based aerobic training program spanned 12 weeks, with participants attending two sessions per week, for a total of 24 sessions. Each 60-min session was structured into three phases: (A) Warm-Up Phase (10 min): This phase involved low-intensity activities such as stretching and flexibility exercises performed at a slow pace to help participants adapt to the rhythm and coordination required; (B) Main Phase (40 min): Participants engaged in moderate-intensity dance routines featuring continuous lower limb movements, trunk mobility, and intermittent arm movements. Each song lasted approximately 4 min, with 2-min breaks between them. The choreography became progressively more complex over the weeks. Dance movements included flexion-extension, abduction-adduction, lateral steps, rotations, rhythm changes, forward and backward movements, foot position changes, heel lifts, and coordinated upper and lower limb motions. Choreographies were carefully selected and sequenced to ensure a logical progression in technical difficulty, incorporating various musical styles such as salsa, rock, rumba, pop, merengue, and bachata. The progression was divided into three stages: (i) Weeks 1–4: The focus was on familiarizing participants with basic dance steps, using simple choreographies with lateral and forward-backward movements; (ii) Weeks 5–8: Complexity increased with the addition of new steps, combinations, and turns that demanded greater coordination and sequential memory; (iii) Weeks 9–12: Advanced choreographies were introduced, featuring complex step sequences designed to enhance memory and cognitive retrieval skills. Participants were also challenged to perform without continuous instructor guidance; (C) Cool-Down Phase (10 min): This final phase consisted of gentle stretching exercises, accompanied by relaxing music, to facilitate a smooth transition to a calm state.

The intervention was conducted under the supervision of a qualified instructor who ensured the safety and effectiveness of the sessions, while considering the specific limitations and individual needs of each participant. Participants were required to complete more than 90% of the sections comprising the intervention to continue in the study.

### 2.4. Outcomes

All data and variables for this study were collected by an independent researcher who was not involved in participant group assignments or the implementation of the intervention. The data gathered included sociodemographic and clinical information, such as age, weight (measured with a precise Tefal digital scale with a capacity ranging from 100 g to 130 kg), height (measured using an Asimed T201-T4 stadiometer), marital status (categories: married, single, separated/divorced, widow), employment status (categories: retired, unemployed, employed), and educational level (categories: no formal education, primary, secondary, university).

#### 2.4.1. Frailty

The “FRAIL” screening questionnaire is a simple tool that comprises 5 questions with yes or no answers [28,29]. These questions explore 5 crucial areas related to frailty: fatigue, endurance, aerobic capacity, comorbidity, and weight loss over the past year. Participants were considered “frail” if they had three or more of the above frailty parameters; those with one or two parameters present were “pre-frail” and those who did not have any were “non-frail”, obtaining a total score ranging between 0 and 5 points.

#### 2.4.2. Cognitive Function

The Mini-Mental State Examination (MMSE) was utilized to evaluate overall cognitive function. This widely recognized tool is designed to identify significant cognitive impairments [30]. The test evaluates five core cognitive domains: attention, calculation, orientation, memory, and language. A maximum score of 30 points represents optimal cognitive performance. The reference scores are categorized as follows: a score of 27 or higher is considered normal, scores of 24 or higher suggest potential cognitive concerns, scores between 12 and 24 indicate impaired cognitive ability, and scores ranging from 9 to 12 are associated with symptoms of dementia.

#### 2.4.3. Cognitive Impairment

The Montreal Cognitive Assessment (MoCA) is a brief test comprising 12 tasks that assess seven distinct cognitive domains: visuospatial and executive functions (including tasks like tracing a figure, copying a cube, and drawing a clock), naming, attention, the ability to recall a series of numbers in reverse order, sustained attention (evaluated through a constant touch task), language skills (such as sentence repetition and verbal fluency), abstract thinking (evaluated with a verbal task), immediate and delayed memory, and orientation. The MoCA has a maximum possible score of 30 points, with scores of 26 or higher considered indicative of normal cognitive function [31].

#### 2.4.4. Verbal Fluency

Verbal fluency was assessed using the Isaac test, in which participants are required to name as many words as possible within a specific semantic category (e.g., animals, fruits, cities, or colors) within a 60-second timeframe. Each category has a maximum score of 10 points, leading to a total possible score of 40 points. A higher score indicates a greater level of verbal fluency [32].

#### 2.4.5. Executive Functions

The Trail Making Test (TMT) was employed to evaluate executive function, emphasizing tasks that require motor coordination and visual skills under time pressure. This test consists of two parts: the first, TMT-A, assesses attention and speed by requiring participants to connect numbered circles in sequential order; the second, TMT-B, involves connecting alternating circles with letters and numbers, specifically assessing executive function [33]. In this context, a longer time to complete the test was interpreted as indicating less efficient performance.

### 2.5. Sample Size Calculation

To obtain the sample size calculation, the Epidat statistical program was used. For the frailty variable, the sample size was estimated to be under a reliability level of 95% and a statistical power of 90%, assuming a standard deviation of 1.2 according to Plaza et al. [34], which indicated that a total of 62 people were required, 31 per group. In addition, a loss to follow-up rate of 20% was considered, resulting in a sample of 72 subjects.

### 2.6. Statistical Analysis

The mean values and standard deviations for each variable of interest were calculated. The Student’s *t*-test for independent samples was used to assess differences between the two study groups. A mixed analysis of variance (ANOVA) was conducted, with the intervention (EG vs. CG) as the between-groups factor and the measurement time (pre-treatment vs. post-treatment) as the within-subject factor. The dependent variables included frailty, cognitive function, verbal fluency, and executive functions. Separate analyses were performed for each dependent variable. An interaction between the treatment (group) and measurement time was explored. Cohen’s d was used to calculate the effect sizes between groups, with values of ≤0.2 indicating a small effect, 0.5 indicating a medium effect, and 0.8 indicating a large effect [35]. A *p*-value of less than 0.05 was considered statistically significant. Statistical analyses were carried out using SPSS statistical software, version 17.0 (SPSS, Inc., Chicago, IL, USA).

## 3. Results

In this study, 36.96% of the participants were men and 63.04% were women. The average age of the participants was 71.83 ± 2.96 years. The majority were retired (72.80%), married (56.50%), and had completed primary education (58.70%) (Table 1). When comparing the groups, no significant differences were found in any of the sociodemographic characteristics.

### 3.1. Frailty

Our results showed statistically significant differences in frailty between the pre- and post-measurements in the training group: t(46) = 9.115, *p* = 0.000, Cohen’s d = 0.60. Additionally, there were significant differences between the two groups in the post-intervention measure: t(90) = 2.953, *p* = 0.004, Cohen’s d = 0.61 (Table 2).

### 3.2. Cognitive Function

Our findings indicated statistically significant differences in cognitive function between the pre- and post-measurements in the treatment/training group: t(46) = −8.094, *p* = 0.000, Cohen’s d = 0.98. Furthermore, significant differences were observed between the two groups in the post-intervention measure: t(90) = −4.663, *p* = 0.000, Cohen’s d = 0.96 (Table 2).

### 3.3. Cognitive Impairment

In terms of cognitive impairment, statistically significant differences were found between the pre- and post-measurements in the treatment/training group: t(46) = −8.535, *p* = 0.000, Cohen’s d = 1.22. Additionally, significant differences were observed between the two groups in the post-intervention measure: t(90) = −6.284, *p* = 0.000, Cohen’s d = 1.29 (Table 2).

### 3.4. Verbal Fluency

Our results showed statistically significant differences in verbal fluency between the pre- and post-measurements in the treatment/training group: t(46) = −7.343, *p* = 0.000, Cohen’s d = 0.61. Furthermore, significant differences were found between the two groups in the post-intervention measure: t(90) = −5.016, *p* = 0.000, Cohen’s d = 1.04 (Table 2).

### 3.5. Executive Functions

Lastly, regarding executive functions, in part A, statistically significant differences were found between the pre- and post-measurements in the treatment/training group: t(46) = −14.947, *p* = 0.000, Cohen’s d = 0.43. Additionally, significant differences were observed between the two groups in the post-intervention measure: t(90) = 19.648, *p* = 0.000, Cohen’s d = 0.64. For part B, statistically significant differences were found between the pre- and post-measurements in the treatment/training group: t(46) = 5.028, *p* = 0.000, Cohen’s d = 0.79, and significant differences between both groups were observed in the post-intervention measure: t(90) = 0.230, *p* = 0.019, Cohen’s d = 0.05 (Table 2).

## 4. Discussion

The objective was to analyze the effectiveness of dance-based aerobic training on frailty and cognitive function in older adults with mild cognitive impairment. The results obtained in this study reflect significant improvements in several parameters related to frailty and cognitive function in older adults with mild cognitive impairment who participated in a dance-based aerobic training program.

Frailty in older people is a multifactorial condition that reflects the intersection between biological aging and socio-environmental risk factors, predisposing individuals to significant adverse consequences for health and quality of life [13]. In our study, the results showed statistically significant differences in frailty before and after the intervention in the training group. In addition, significant differences were observed between the groups in the post-intervention measures. These findings suggest that a dance-based aerobic training program can effectively reduce frailty levels in older adults with mild cognitive impairment. Existing literature supports and extends these findings, such as the study by Cadore et al. [35] in which it was observed that multicomponent interventions that include both strength and aerobic training can be especially beneficial in reducing frailty in older people. Furthermore, a study by Lafortune et al. [36] found that regular exercise, including dancing, can significantly improve indicators of physical frailty and stress resistance in older adults. On the other hand, a study by Tarazona-Santabalbina et al. [37] suggests that even lower intensity, but regular, exercises can be effective in improving functionality and reducing frailty in older adults, highlighting the importance of personalizing exercise interventions according to abilities and individual needs. These results encourage the implementation of well-structured exercise programs that not only focus on improving overall physical fitness, but are also designed to improve cognitive ability and overall quality of life in this population.

Cognitive function is essential for quality of life in older adults, including abilities such as memory, reasoning, and information processing, the decline of which is often linked to aging and conditions such as mild cognitive impairment (MCI) [38]. In our study, statistically significant improvements in cognitive function were observed in the treatment/training group. These findings indicate that dance-based aerobic training may be an effective method to improve cognitive function in older adults with MCI. Existing literature offers support for these results, such as the study by Lautenschlager et al. [39] who found that older adults with mild cognitive impairment who participated in a physical exercise program showed improvements in cognitive function compared to those who did not participate in exercise. Similarly, the meta-analysis by Chan et al. [40] examined the effect of exergaming interventions on cognitive functions and found improvements in executive function and memory in older adults with mild cognitive impairment (MCI), including delayed recall and verbal fluency. In addition, interventions that combine physical and mental elements such as Tai Chi and yoga have also proven to be beneficial. A study by Lam et al. [41] suggests that Tai Chi significantly improves both cognitive function and physical mobility in older adults with MCI. These interventions suggest that the combination of physical and cognitive stimuli may be particularly potent in mitigating the effects of cognitive decline. Compared to these studies, our results confirm and expand the evidence that complex and stimulating physical activities such as dance not only serve to maintain physical fitness but also to improve various areas of cognitive function. This finding is particularly relevant, since dancing involves learning steps and sequences, which can stimulate memory, concentration, and coordination, key elements in general cognitive functionality.

Mild cognitive impairment (MCI) is an intermediate stage between the cognitive decline expected with normal aging and the more serious disorders associated with dementia, such as Alzheimer’s disease. Individuals with MCI may experience a decline in cognitive abilities, such as memory, language, and judgment, which can impact their daily lives. Therefore, it is essential to identify interventions that can mitigate or reverse these effects. In our study, participants who received dance-based aerobic training showed significant improvements in cognitive decline. However, despite these improvements, their MMSE score indicated that they remained within the range corresponding to a diagnosis of mild cognitive impairment. These findings highlight that dance can be an effective tool for improving cognition in older adults with MCI. The existing literature reinforces the idea that physical and cognitive interventions can benefit this population. A study by Lautenschlager et al. [39] showed that physical exercise could improve cognitive function in older adults with MCI, suggesting that regular physical activity could be a promising strategy to mitigate cognitive decline. Similarly, a study by Suzuki et al. [42] found that the combination of aerobic exercise and resistance training had positive effects on memory and other cognitive functions in older adults with MCI. Additionally, interventions such as yoga and tai chi, which combine physical and meditative components, have also been shown to be effective. For example, a study by Eyre et al. [43] evaluated the impact of yoga and meditation in older adults with MCI and reported improvements in memory and other cognitive functions, suggesting that these practices may offer additional cognitive benefits. Comparing our results, it is clear that while these studies support the usefulness of various forms of exercise in improving cognition in older adults with MCI, our study highlights dance as a particularly effective intervention, possibly due to its comprehensive nature requiring coordination, rhythm, sequence memory, and social interaction.

Verbal fluency is a critical cognitive ability that involves the ease of generating words and forming sentences and is essential for effective communication. In older adults, verbal fluency may be affected by cognitive decline, underscoring the importance of identifying interventions that can sustain or improve this function. In our study, verbal fluency showed significant improvements both in internal measurements of the intervention group and in comparisons with the control group. These results suggest that a dance-based aerobic training program may be effective in improving verbal fluency in older adults with mild cognitive impairment. Previous studies have examined various interventions that could positively influence verbal fluency in this population. For example, a study by Smith-Ray et al. [44] evaluated the effects of physical exercise on cognition in older adults and found improvements in verbal fluency in those who participated in regular physical activities. This finding is in line with our study, suggesting that physical exercise may have a positive impact on this area of cognitive function. Furthermore, a study by Wu et al. [45] investigated the effects of dance on the cognitive function of older adults, reporting improvements in verbal fluency and other cognitive functions, which supports the idea that dance, being an activity that combines physical exercise, socialization, and coordination, can be particularly beneficial for verbal fluency. On the other hand, interventions that include specific cognitive components, such as language skills training and speech therapy, have also been shown to be effective. A study by Nocera et al. [46] showed that a combined program of physical exercise and cognitive training improves verbal fluency in older adults with cognitive impairment. These studies suggest that a combined approach may be ideal for maximizing verbal fluency benefits. In summary, our study adds to the growing evidence that aerobic exercise, and specifically dance, may be an effective intervention to improve verbal fluency in older adults with mild cognitive impairment. Regular physical activity, particularly that requiring coordination and rhythm, appears to offer significant benefits for this crucial cognitive function.

Executive functions are key mental skills that include planning, decision-making, problem-solving, impulse control, and multitasking. These functions are crucial to daily life and are often compromised in older adults, especially those with mild cognitive impairment (MCI). Effective interventions that promote the improvement of these skills are essential to maintaining autonomy and improving the quality of life in this population. In our study, significant improvements in executive functions were observed in participants who received dance-based aerobic training. These findings suggest that dance may be an effective intervention to improve executive functions in older adults with MCI. The literature supports the effectiveness of physical exercise in improving executive functions in this population. A study by Baker et al. [47] investigated the effects of aerobic exercise on cognitive function in older adults with MCI and found improvements in executive functions, but unlike our study, they did not find significant improvements in memory. Similarly, a study by Erickson et al. [48] demonstrated that aerobic exercise increases the size of the hippocampus and improves performance in tasks that require high executive demand. Additionally, interventions that combine physical exercise with cognitively challenging activities, such as strategy games and puzzles, have also been shown to be beneficial. A study by Chan et al. [40] explored the impact of exergaming on the cognitive function of older adults and found that those who participated in activities that combined physical exercise and cognitive challenges showed improvements in executive function. Comparing these studies with our findings, it is evident that dance, by integrating physical, cognitive, and social aspects, offers an attractive and effective means for improving executive functions in older adults. Regular physical activity, especially that which includes rhythmic components, step memorization, and timing, appears to provide significant cognitive stimuli necessary to enhance executive ability in this demographic group.

Despite the positive findings, it is important to recognize some limitations of our study. The lack of long-term follow-up makes it impossible to determine the duration of the training effects. Future studies should consider including a larger number of participants and extending the follow-up period to evaluate the persistence of cognitive and physical benefits.

## 5. Conclusions

This study provides significant evidence that dance, as a form of aerobic exercise, not only improves physical frailty but also contributes to optimizing cognitive function in older adults with mild cognitive impairment. In particular, significant improvements were observed in executive functions, verbal fluency, global cognitive impairment, and general cognitive function. The results underline the importance of integrating physical and mental stimulation interventions in care programs for this population, promoting a comprehensive approach to healthy aging. Furthermore, these findings suggest future lines of research and development in clinical practice, focused on maximizing the benefits of combined interventions to prevent or delay age-related decline.

## Figures and Tables

**Table 1:** Preintervention sociodemographic and clinical characteristics of the participants as a whole and by group.

		Total (*n* = 92)	Experimental (*n* = 47)	Control (*n* = 45)	*p*-Value
Age		71.83 ± 2.96	71.43 ± 2.97	72.24 ± 2.92	0.783
Sex	Male	34 (36.96)	18 (52.90)	16 (47.10)	0.672
	Female	58 (63.04)	29 (50.00)	29 (50.00)	
Occupational Status	Retired	67 (72.80)	35 (52.20)	32 (47.80)	0.586
	Worker	0 (0.00)	0 (0.00)	0 (0.00)	
	Stopped	25 (27.20)	12 (48.00)	13 (52.00)	
Civil status	Single	13 (14.10)	7 (53.80)	6 (46.20)	0.710
	Married	52 (56.50)	26 (50.00)	26 (50.00)	
	Divorced/Separated/Widowed	27 (29.30)	14 (51.90)	13 (48.10)	
Educational status	Without studies	14 (15.20)	8 (57.10)	6 (42.90)	0.090
	Primary studies	54 (58.70)	31 (57.40)	23 (42.60)	
	Secondary studies	16 (17.40)	5 (31.20)	11 (68.80)	
	University studies	8 (8.70)	3 (37.50)	5 (62.50)	
Frailty		2.43 ± 1.74	2.43 ± 1.77	2.44 ± 1.74	0.908
Cognitive function		21.42 ± 1.74	21.49 ± 1.74	21.36 ± 1.76	0.936
Cognitive impairment		21.39 ± 1.05	21.36 ± 1.07	21.42 ± 1.03	0.769
Verbal fluency		26.68 ± 2.46	26.81 ± 2.49	26.56 ± 2.45	0.807
Executive functions, part A		105.76 ± 41.45	115.28 ± 40.04	95.81 ± 41.97	0.990
Executive functions, part B		191.63 ± 81.39	222.11 ± 68.81	159.80 ± 81.98	0.423

Quantitative variables are presented as mean and standard deviation. Qualitative variables are presented as frequency and percentage. BMI: Body Mass Index.

**Table 2:** Effects of a dance-based aerobic training program on frailty and cognitive capabilities.

	EG (*n* = 47)		CG (*n* = 45)		Group			Time			Group × Time		
	Pre	Post	Pre	Post	F(90)	*p*-Value	η^2^	F(90)	*p*-Value	η^2^	F(90)	*p*-Value	η^2^
Frailty	2.43 ± 1.77	1.49 ± 1.33	2.44 ± 1.74	2.49 ± 1.88	2.321	0.131	0.025	16.017	0.000	0.151	19.369	0.000	0.177
Cognitive function	21.49 ± 1.74	23.04 ± 1.37	21.36 ± 1.76	21.51 ± 1.77	7.366	0.008	0.076	27.519	0.000	0.234	18.410	0.000	0.170
Cognitive impairment	21.36 ± 1.07	22.53 ± 0.83	21.42 ± 1.03	21.07 ± 1.36	17.620	0.000	0.164	7.117	0.009	0.073	24.963	0.000	0.217
Verbal fluency	26.81 ± 2.49	28.23 ± 2.18	26.56 ± 2.45	25.93 ± 2.22	8.007	0.006	0.082	4.733	0.032	0.050	30.759	0.000	0.255
Executive functions, part A	115.28 ± 40.04	97.64 ± 40.23	95.82 ± 40.98	123.18 ± 39.13	0.136	0.714	0.002	14.548	0.000	0.138	30.966	0.000	0.775
Executive functions, part B	222.11 ± 68.81	167.45 ± 68.84	159.80 ± 81.98	170.76 ± 69.28	4.736	0.031	0.050	10.378	0.002	0.103	23.394	0.000	0.206

Quantitative variables are presented as mean and standard deviation. EG: experimental group; CG: control group.

## Data Availability

Data are contained within the article.
